# Nanomaterials towards Biosensing of Alzheimer’s Disease Biomarkers

**DOI:** 10.3390/nano9121663

**Published:** 2019-11-22

**Authors:** Pedro Carneiro, Simone Morais, Maria Carmo Pereira

**Affiliations:** 1 LEPABE–Laboratory for Process Engineering, Environment, Biotechnology and Energy, Faculty of Engineering, University of Porto, Rua Dr. Roberto Frias, 4200-465 Porto, Portugal; carneiro.pedrojs@gmail.com (P.C.); mcsp@fe.up.pt (M.C.P.); 2 REQUIMTE–LAQV, Instituto Superior de Engenharia do Porto, Instituto Politécnico do Porto, R. Dr. António Bernardino de Almeida 431, 4200-072 Porto, Portugal

**Keywords:** nanomaterials, electrochemical, optical, immunosensor, DNA sensor, biosensor, gold nanoparticles, carbon nanotubes, graphene, polymers

## Abstract

Alzheimer’s disease (AD) is an incurable and highly debilitating condition characterized by the progressive degeneration and/or death of nerve cells, which leads to manifestation of disabilities in cognitive functioning. In recent years, the development of biosensors for determination of AD’s main biomarkers has made remarkable progress, particularly based on the tremendous advances in nanoscience and nanotechnology. The unique and outstanding properties of nanomaterials (such as graphene, carbon nanotubes, gold, silver and magnetic nanoparticles, polymers and quantum dots) have been contributing to enhance the electrochemical and optical behavior of transducers while offering a suitable matrix for the immobilization of biological recognition elements. Therefore, optical and electrochemical immuno- and DNA-biosensors with higher sensitivity, selectivity and longer stability have been reported. Nevertheless, strategies based on the detection of multiple analytes still need to be improved, as they will play a crucial role in minimizing misdiagnosis. This review aims to provide insights into the conjugation of nanomaterials with different transducers highlighting their crucial role in the construction of biosensors for detection of AD main biomarkers.

## 1. Introduction

Neurodegenerative diseases (NDDs), whose prevalence is rapidly escalating, mainly in association with the increase of the elderly population, are a heterogeneous group of disorders characterized by the progressive degeneration of the structure and function of the central and/or peripheral nervous systems [1,2,3]. NDDs have become a major health problem for the world’s population, with a tremendous financial impact associated with medical care for both families and society [4,5,6].

Alzheimer’s disease (AD) is the most common NDD, as well as the most common form of dementia. Nowadays, this disease affects approximately 40 million people, but its numbers are predicted to escalate in the upcoming decades [7,8,9,10]. AD pathophysiology involves a combination of processes that ultimately lead to loss of synaptic integrity, effective neural connectivity and progressive neurodegeneration [7,8,10,11]. It is believed that the accumulation of amyloid β protein (Aβ) in extracellular deposits is the primary event that eventually drives the formation of intraneuronal inclusions of hyperphosphorylated tau protein, both accompanied by processes of inflammation and oxidative stress [7,8,10]. In addition, despite the presence of Aβ aggregates being considered the key pathological hallmark of AD, studies have revealed that Aβ oligomers may be its most toxic form, leading to synaptic dysfunction [7,8,12]. Clinical application of these biomarkers can be very challenging, as the most commonly reported cut-off values in cerebrospinal fluid (CSF) are 500–700 pg mL^−1^ (110–155 pM) [13] and 195 pg mL^−1^ (4.3 pM) [14] for Aβ42 and tau protein, respectively. Moreover, studies have also focused on understanding the genetic components that influence the risks and outcomes of neurological disease identifying the isoform apolipoprotein E4 (ApoE4) as a major genetic risk factor for AD and decreased age of onset, as well as recognizing the protective role of the isoform ApoE2 against the disease [15,16,17].

During the last century, life expectancy has increased as a result of major improvements in worldwide health. Nevertheless, this fact can also increase the prevalence of AD as the risk of incidence of this disease increases dramatically with age which accentuates the need to develop new methodologies for its treatment, diagnosis and prevention [2,6]. In this context, it is highly desirable to develop multiplexed devices capable of screening various biomarkers simultaneously promoting a rapid, low-cost and reliable diagnosis in addition to contributing to a better understanding of the molecular mechanisms and cascade of events underlining the disease pathophysiology [18,19].

Over the last decade, the number of studies concerning the development of rapid and easy-to-use platforms for the detection of analytes based on biosensing technology has significantly increased, as these devices display a wide range of benefits, such as high sensitivity and selectivity without the need for large sample volumes. A biosensor is defined by IUPAC as an integrated transducer-receptor device, which combines a biological recognition element with a transducer capable of converting the biological response into quantitative or semi-quantitative analytical information [20,21]. Accordingly, biosensors can be classified in terms of their biological recognition elements and transduction principle [21,22,23]. Immunosensors and DNA sensors are affinity ligand-based biosensing devices that involve the transduction of immunochemical reactions benefiting from the highly specific interaction between antibody–antigen and DNA strands, respectively [21,22,23,24,25]. Development of biosensors with high sensitivity through the enhancement of their active surface area, (electro)chemical activity and conductivity or optical properties is one of the major challenges of the biosensing field [21,25,26,27,28]. This way, the use of nanomaterials has been regarded as a crucial parameter for enhancing biosensor analytical performance. In fact, nanomaterials have now become an integral part of every biosensing platform, promoting an increase in sensitivity, which is reflected in their capability to lower detection limits by several orders of magnitude [21,25,26,27,28]. The introduction of nanomaterials into biosensing platforms and the optimization of their interaction with biological recognition elements will contribute to the development of highly sensitive and specific point-of-care technology (POCT). The aim of POCT is the conception of high-performance devices with low system complexity, ensuring sensitive analysis in non-laboratory and resource-limited settings with user-friendly equipment while minimizing the analysis time [18,29,30]. 

The goal of this review is to present an overview of the most frequently used nanomaterials in the development of immunosensors and DNA sensors for the quantification of AD biomarkers covering the period from 2008 to 2018. In the following sections, the role of nanomaterials in the structure of biosensors is critically discussed, while their interactions with biological recognition elements, the strategy for transducing the bioaffinity event and the analytical performance of the biosensors are highlighted. Finally, this review brings into focus future perspectives on the impact of nanomaterials in AD biosensing.

The available scientific literature within the last decade (2008–2018) was searched in the Thomson Reuters ISI Web of Knowledge, Science Direct, PubMed and Google Scholar databases by combining at least two of the following keywords: biosensor/electrochemical immunosensor/optical immunosensor/DNA sensor in conjunction with Alzheimer’s disease/Amyloid beta/Tau protein/Apolipoprotein E.

## 2. Nanomaterials in Biosensing of Neurodegenerative Disease Biomarkers

In recent decades, novel modified transducers have been developed based on the unique properties of nanoscale materials and the ability to tailor their size and structure. As a result of quantum-size effects, nanomaterials exhibit great electronic, mechanical, thermal and optical properties and are recognized as one of the most attractive ways to promote the design of biosensors with enhanced analytical performance [21,31,32,33]. Integration of nanomaterials into biosensors has been found to improve the conductivity and catalytic activity of the transducer while favoring the immobilization of a large amount of biological recognition elements, as a result of their high surface area, in addition to improving the accessibility of specific analytes to these elements [26,28,31,32,33,34]. In fact, functionalization of nanomaterials has contributed to the development of highly sensitive and selective bioassays and biosensors for nucleic acids and proteins by integrating the biological recognition elements with the components of the various transduction mechanisms [26,27,34,35]. In this regard, considerable attention has been devoted to the immobilization of biological recognition elements as this aspect will have an impact on detection sensitivity, reproducibility and robustness, among other analytical parameters [25,27,33,36]. The performance of a biosensor is predominantly dependent on the binding affinity and specificity of binding molecules, their coating density onto the transducer’s surface and, finally and most importantly, the orientation of the biological recognition elements after immobilization, which should retain their full biological activity by ensuring that its binding sections remain intact and accessible while also providing an effective electronic connection between the redox active sites in the biomolecules and the transducer’s surface [26,32,37,38,39].

**Figure 1:**
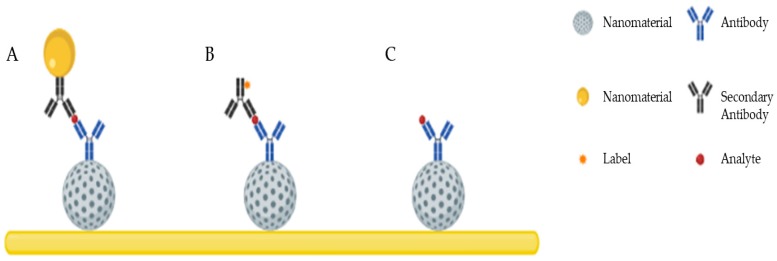
Schematic illustration of the most common detection schemes developed using nanomaterials: (**A**) sandwich assay with secondary antibodies conjugated with nanomaterials and without labels; (**B**) sandwich assay with labeled secondary antibodies; (**C**) direct detection.

The most frequently applied nanomaterials in the development of immunosensors and DNA biosensors are carbon materials such as graphene and carbon nanotubes (CNTs), gold nanoparticles (AuNPs) and polymers, all having unique and specific properties applicable in the development of novel transduction schemes. Other nanomaterials such as silver (AgNPs) and magnetic nanoparticles, dendrimers and quantum dots (QDs) are also used, but to a lesser extent. These nanomaterials have most commonly been reported for the development of biosensors performing detection via sandwich immunoassays/DNA assays or through direct detection (Figure 1). 

### 2.1. Carbon Nanomaterials

Carbon-based materials including CNTs, graphene, fullerenes, carbon fibers, graphene quantum dots and carbon dots have been receiving a lot of attention in the development of biosensing analytical tools [37,40,41,42,43], with CNTs [44,45,46,47] and graphene [48,49,50] being the most commonly applied carbon nanomaterials for the development of biosensors for the detection of AD biomarkers (Table 1). Although the specific characteristics of carbon nanomaterials vary between them, their most prominent advantages are reflected in their electrochemical activity, electrical conductivity, large surface area, high surface-to-volume ratio, ease of functionalization, biocompatibility and anti-fouling effect [27,33,35,37,40,42]. 

#### 2.1.1. Carbon Nanotube-Based Biosensors

**Figure 2:**
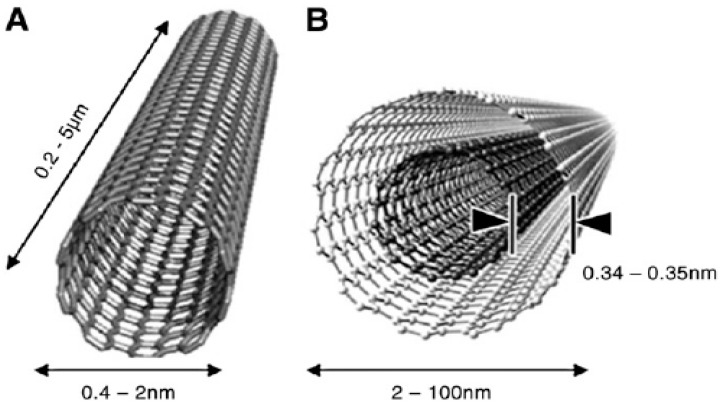
Structure of single-walled carbon nanotubes (SWCNTs) (**A**) and multi-walled carbon nanotubes (MWCNTs) (**B**) (Reproduced with permission from [57]. Elsevier, 2010).

CNTs are cylindrical large molecules consisting of a hexagonal arrangement of hybridized carbon atoms, which can be classified as single-walled carbon nanotubes (SWCNTs), composed of a single graphite sheet rolled into a seamless hollow nanoscale tube, and multi-walled carbon nanotubes (MWCNTs), characterized by the presence of multiple concentric tubes encircling one another [41,51,52] (Figure 2). SWCNTs present a diameter in the range of 0.4–2 nm, while MWCNTs, depending on the number of layers, can display a diameter in the range of 2–100 nm, with the distance between each layer being approximately 0.34 nm [26,33,41,52,53,54,55,56,57]. Since their discovery in 1991 [58], they have increasingly attracted research interest due to their high surface-to-volume ratio, exceptional electronic properties, and the presence of edge-plane-like defects which make them very interesting for biosensing applications [26,33,41,52,56,59]. In addition, another major advantage of CNTs is that they can be easily functionalized with different chemical groups through covalent and non-covalent bonds, which will further promote the immobilization of biomolecules or organic molecules [26,33,37,56]. 

**Figure 3:**
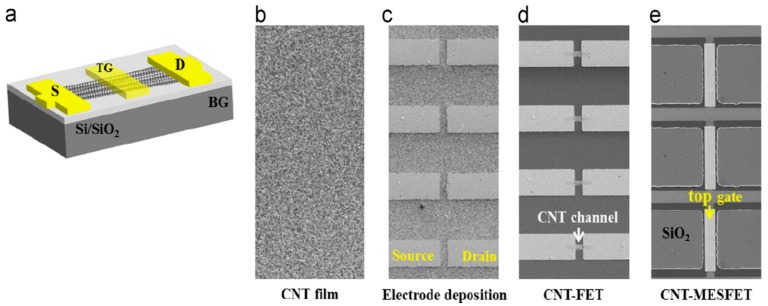
(**a**) Schematic diagram of a carbon nanotubes (CNTs)-metal semiconductor field effect transistor structure device. (**b**–**e**) Fabrication procedure of the metal semiconductor field effect transistor structure device (Reproduced with permission from [44]. Elsevier, 2013).

Oh et al. [44] developed a SWCNTs film-based biosensor with a metal semiconductor field effect transistor structure (CNTs-MESFET) (Figure 3). A gold strip was deposited in the middle of the CNTs channel, leading to the formation of a potential energy barrier (Schottky barrier) between both materials [44]. This way, any changes in the CNTs channel covered with the gold strip would cause a variance in the CNTs channel conductance, enabling the detection of the target analytes in real-time. In this work, antibodies were immobilized on the gold surface after its functionalization with *Escherichia coli* outer membrane with autodisplayed z-domains of protein A. By controlling the orientation of antibodies upon their immobilization, the authors were able to attain higher sensitivities while also achieving effective blocking towards unspecific binding, which was mainly attributed to the highly negative charge of the outer membrane. In this way, the CNT-MESFET biosensor enabled real-time detection of Aβ42 at levels as low as 1 pg mL^−1^ in human serum, while a linear relation was observed in the range of 10^−12^–10^−9^ g mL^−1^ [44]. 

In a different work, Yu et al. [45] used SWCNTs to develop a ratiometric electrochemical biosensor for the simultaneous determination of Cu^2+^ and Aβ based on a glassy carbon electrode (GCE). To produce a stable colloidal suspension and favor the stability and electron transfer of CNTs, SWCNTs were functionalized with poly (diallyldimethylammonium chloride) (PDDA) and 2,2′-azinobis-(3-ethylbenzothiazoline-6-sulphonate) (ABTS) [45]. A homogeneous and dense distribution of surface functional groups was achieved by the non-covalent functionalization of SWCNTs with PDDA, which revealed itself to be essential for further assembly of negatively charged ABTS and recognition elements. The inclusion of ABTS enhanced the sensitivity of the bioelectranalytical system, while also improving its accuracy by acting as an inner reference molecule. This system was first assembled for sensitive and accurate determination of Cu^2+^, through its electroreduction, by immobilizing neurokinin B (NKB) for its recognition. Furthermore, due to strong complexation of Cu^2+^ with Aβ, Aβ42 could also be detected by the same biosensor as upon its addition, Cu^2+^ would be released from the previously formed Cu(NKB)_2_ complex causing a current signal reduction [45]. For that reason, both Cu^2+^ and Aβ42 were easily monitored by the same system attaining limits of detection (LOD) of 0.04 μM and 0.5 ng mL^−1^, respectively [45]. Despite exhibiting the highest LOD in Table 1 for Aβ42 determination, Yu et al. [45] were able to verify an increase in the levels of Cu^2+^ in plasma of AD rats while attaining recoveries of 97–110% in hippocampus of rats spiked with Aβ42. 

For the determination of Aβ42/Aβ40 levels in cerebrospinal fluid and targeted brain tissue of AD rats, Yu et al. [46] developed an electrochemical affinity biosensor based on MWCNTs modified with AuNPs. MWCNTs were implemented with an enhancement role due to their excellent conductivity and electrochemical activity, causing an increase of the charging currents when compared to the bare GCE. On the other hand, AuNPs played a dual role by contributing to the enhancement of charging current while offering a suitable environment for the immobilization of the biological recognition element, which in this case was a molecular secretory protein with high affinity towards soluble Aβ peptide designated gelsolin [46]. In this way, the developed electrochemical biosensor was based on a sandwich-type assay which consisted of a GCE modified with MWCNTs and AuNPs where gelsolin was further immobilized for the biological recognition process. In addition, a bioconjugate of AuNPs functionalized with gelsolin and horseradish peroxidase was prepared to complete the sandwich assay attaining through this process a LOD of 28 pM being the Aβ40 and Aβ42 peptides in a 6:1 concentration ratio [46]. In addition, applying the developed biosensor in real samples it was possible to observe a clear variation in the CSF and brain tissue levels of Aβ42/Aβ40 between normal and AD rats. Conversely, even though the developed biosensor was based on a bioreceptor with high affinity towards Aβ, its capability to distinguish between Aβ42 and Aβ40 was not evaluated. Moreover, the biosensor was only evaluated for a two-week stability period.

**Figure 4:**
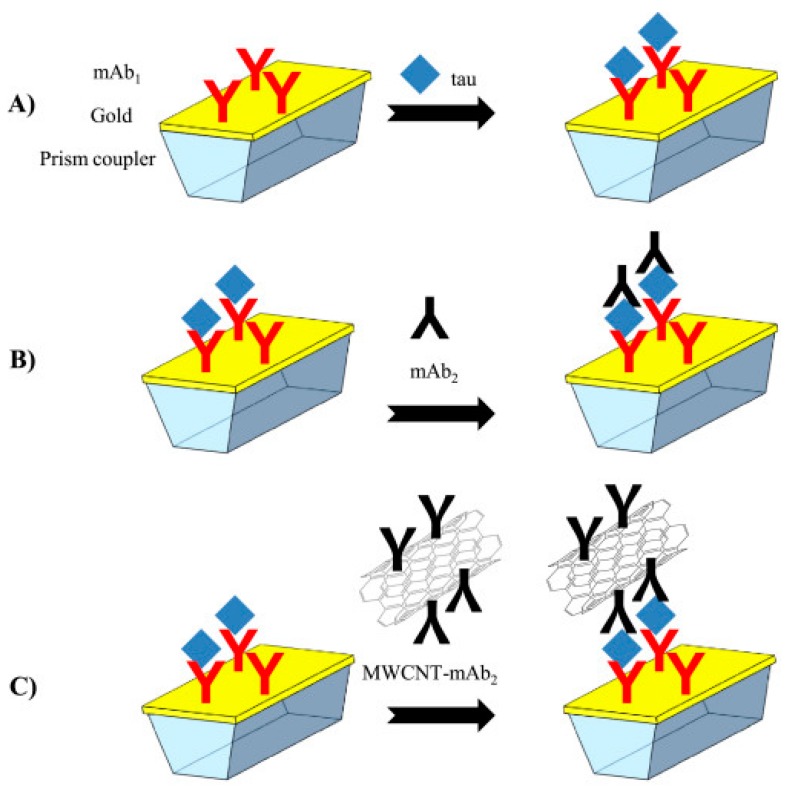
Design of surface plasmon resonance (SPR) assays for tau quantification: (**A**) direct approach; (**B**) sandwich assay; (**C**) sandwich assay with secondary antibodies coupled with multi-walled carbon nanotubes (MWCNTs) (Reproduced with permission from [47]. Elsevier, 2017).

In the last reported study using CNTs for biosensing of AD biomarkers, Lisi et al. [47] developed a biosensor for tau protein detection using a layer-by-layer approach for amplification of the surface plasmon resonance (SPR) signal (Figure 4). Here, MWCNTs were oxidized in order to overcome their hydrophobic nature and promote their stability in aqueous solutions in addition to providing carboxylic groups for their covalent functionalization with secondary antibodies against tau protein via amino coupling reaction. MWCNTs–antibody conjugate was essential for the achievement of the sandwich bioassay, contributing to the increase of SPR signal by 10^2^ fold when compared to direct detection and conventional unconjugated sandwich (Figure 4). In fact, MWCNTs functionalized with secondary antibodies were used as mass enhancers, providing a consistent refractive index change [47]. Applying this system, and under the optimal conditions, a dose–response relation was displayed, enabling the determination of tau concentrations in the range 125–1000 pM in which an exponential behavior was observed while exhibiting good reproducibility and selectivity [47]. Nevertheless, in order to be applicable in a clinical context, some parameters need to be further evaluated, such as its stability over time and analytical performance in real samples, as well as lowering the reported LOD.

#### 2.1.2. Graphene

**Figure 5:**
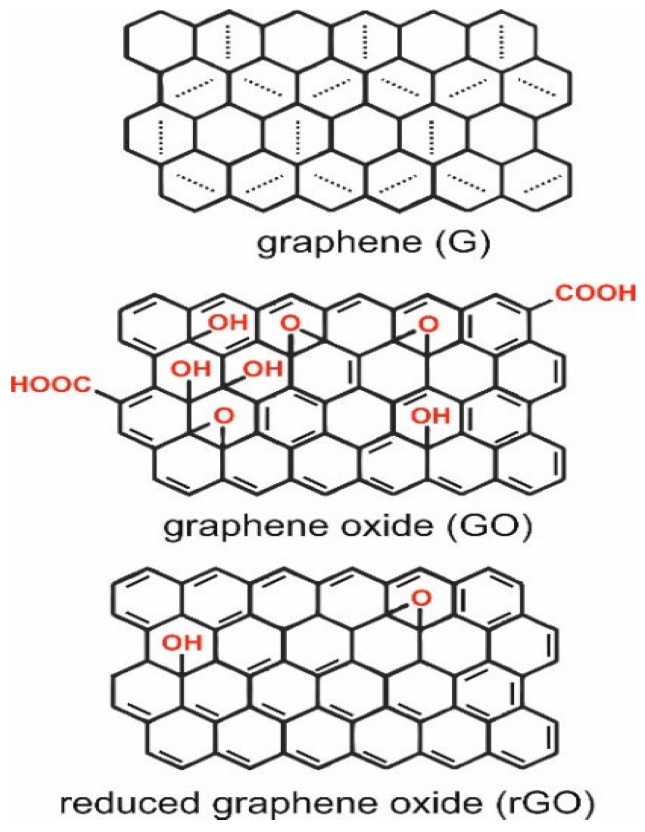
Structure of graphene, graphene oxide and reduced graphene oxide [66].

Graphene is a planar sheet of carbon atoms arranged into a rigid honeycomb structure, and like CNTs, the carbon bonds are sp^2^-hybridized [60,61,62,63,64,65]. As a result of its electron configuration, graphene exhibits large surface area, high mechanical strength, high electrical conductivity, high elasticity and thermal conductivity [60,61,62,63,64,66]. Graphene oxide (GO) and reduced GO (rGO) are derivatives of graphene with a vast applicability in the biosensing field (Figure 5). Graphene can be easily functionalized into GO containing various oxygen functional groups, such as epoxide, carbonyl, carboxyl and hydroxyl groups [62,63,66] (Figure 5). These hydrophilic groups make GO more soluble in water, while exhibiting better selectivity towards functionalization with biomolecules, which are highly important features in biosensor applications [60,62,63]. On the other hand, rGO is the form of GO that is processed by chemical, thermal and other procedures which will ultimately influence its composition and properties [62]. The reduction process will reduce the oxygen content while introducing structural defects that will contribute to high thermal conductivity as the electrochemistry of graphene sheets occurs at the edges and defects (where heterogeneous electron transfer is fast) and not at the basal plane [61,62,63,66] (Figure 5). Usually, rGO presents advantages over graphene and GO for application in biosensing technology as it combines some of the negatively charged groups of GO along with the excellent conductive properties of graphene [65,67].

Graphene has been used in three different ways for the development of electrochemical immuno- and DNA biosensors for determination of AD biomarkers, namely, Aβ and ApoE, respectively. Two works performed determination of ApoE through DNA detection, with Mars et al. [48] using graphene quantum dots for a dual biosensing platform with electrochemical and fluorescence determination, while Wu et al. [49] conjugated graphene with mesoporous silica for the development of a hybrid nanomaterial that served as the basis for ratiometric determination. Finally, Kurkina et al. [50] applied rGO for the development of a FET electrochemical immunosensor for Aβ determination. 

The biosensing platform developed by Mars et al. [48] was based on indium tin oxide (ITO) modified in a layer-by-layer approach with graphene quantum dots (GQDs) and electropolymerized curcumin for detection of ApoE4 DNA. GQDs formed a three-dimensional wrinkled layer on the ITO surface that enhanced the electron transfer rate and the electroactivity of curcumin as a result of the abundant presence of active carboxylic sites allowing the creation of hydrogen bonds with curcumin molecules [48]. The analytical signal was obtained from the quenching of curcumin signal after the hybridization of the DNA complex, which was promoted through the immobilization of a DNA probe on the modified ITO via EDC/NHS chemistry. Applying the proposed biosensor, a LOD of 2.18 pg mL^−1^ through amperometric measurements was attained. On the other hand, through analysis of the fluorescence response of the modified ITO, an estimated 12.4 pg mL^−1^ LOD was achieved, which could be related to the quenching of the photoelectron transfer process induced by the formation of the DNA complex [48]. In addition, the biosensor displayed good selectivity and reproducibility, while exhibiting good analytical behavior when exposed to human plasma. Also, concerning the detection of ApoE gene, Wu et al. [49] developed a ratiometric electrochemical platform based on a GCE modified with graphene and mesoporous silica hybrid nanomaterials (GSHs). Electroactive molecules with distinct separate oxidation potentials, specifically methylene blue (MB) and ferrocenecarboxylic acid (Fc), were employed as the indicator for target and built-in control, respectively. GSHs, produced via a reducing process with mesoporous silica homogeneously coated on the surface of graphene sheets, acted as reservoirs for the electroactive MB while the Fc molecules were covalently conjugated with the produced nanomaterials via a carbodiimide-mediated approach [49]. For the production of the analytical signal, a duplex DNA probe was firstly immobilized on the GSHs surface, promoted by the immobilization of a single-stranded DNA through a 4-maleimidobutyric acid *N*-hydroxysuccinimide (NHS) ester, caging the electroactive MB which was only released upon addition of the target DNA generating a measurable “on-off” current [49]. GSHs combined the advantages of both graphene and mesoporous silica into a single hybrid nanostructure, promoting a faster electron transfer process, due to its good conductivity and large surface area, while encapsulating electroactive molecules [49]. With this electrochemical configuration, ApoE DNA levels as low as 10 fM were able to be determined.

**Figure 6:**
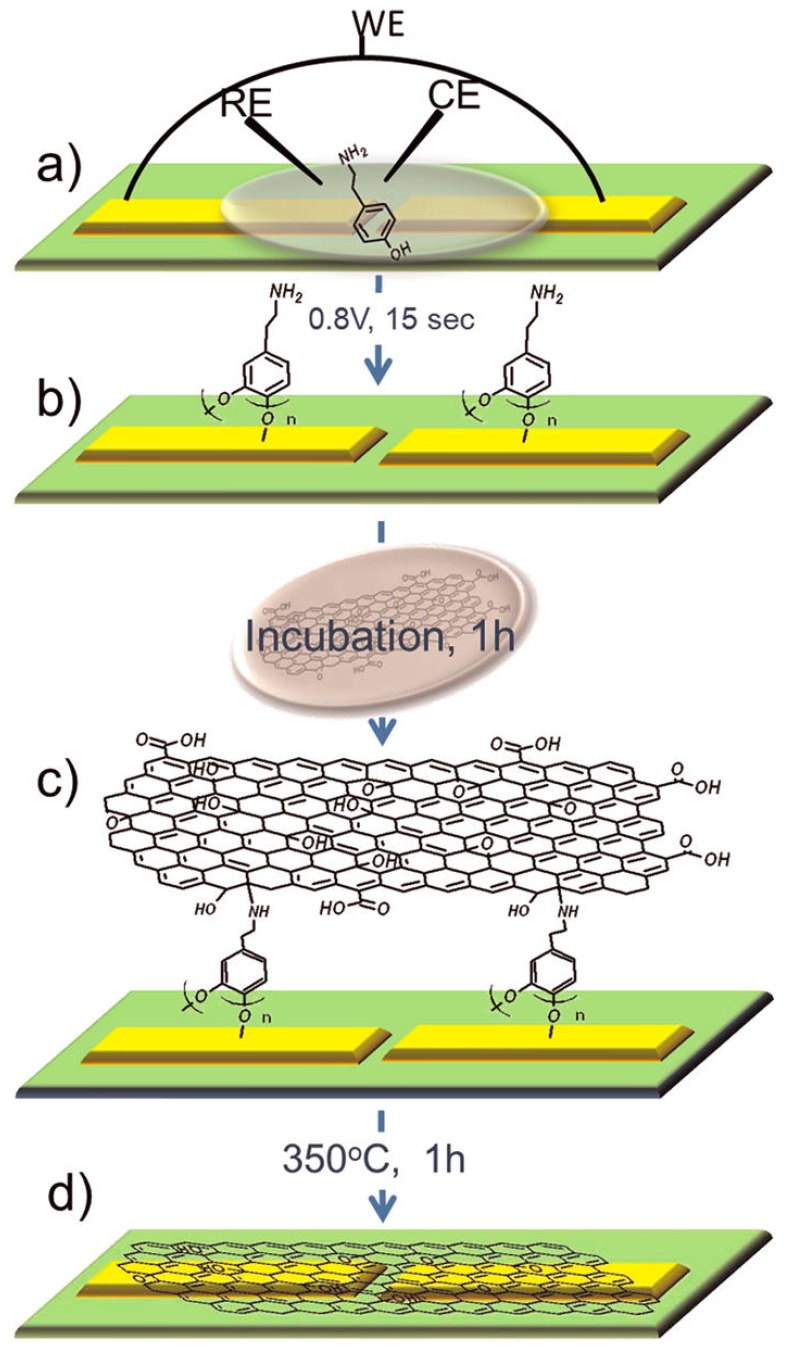
Scheme of the chemical anchoring protocol: (**a**) electrochemical functionalization of Pt electrodes with tyramine; (**b**) coating with polytyramine on the electrode surface; (**c**) incubation of the chip in a Graphene oxide (GO) solution; (**d**) annealing in argon at 350 °C. WE = working electrode, CE = counter electrode, RE = reference electrode (Reproduced with permission from [50]. American Chemical Society, 2012).

Regarding the determination of Aβ, Kurkina et al. [50] applied a rGO-FET liquid-gated configuration as an electrochemical immunosensor (Figure 6). This work described a chemical process for the development of a FET device from GO. The procedure involved amino functionalization of photolithographically patterned electrodes that reacted with the functional groups on the GO surface in order to promote its selective immobilization at the electrode locations. GO was then thermally reduced, displaying a graphene-like field-effect behavior while promoting an appropriate environment for the biorecognition event [50]. The proper orientation of antibodies against Aβ on the rGO surface was ensured through its conjugation with *Staphylococcus aureus* protein A. Applying impedance spectroscopy analysis and taking advantage of the high sensitivity of rGO to chemical changes in their surroundings, a 1 fM Aβ concentration was determined [50]. 

The biosensors developed by Wu [49] and Kurkina [50], despite displaying extremely good analytical behavior and low LODs, failed to address the parameters of selectivity, reproducibility and performance in real samples. Moreover, all three reported studies using graphene for the development of highly sensitive biosensors further need to be evaluated in terms of stability.

### 2.2. Nanoparticles

Nanoparticles with varied configurations and properties have been the most frequently used nanomaterials for the development of novel immuno- and DNA-biosensors [68,69]. Owing to their small size and high surface area, nanoparticles offer unique chemical, physical and electronic properties that are advantageous for the development of high-performance biosensors either by using them as amplifying labels for signal enhancement or by working as an appropriate platform for the immobilization of biological recognition elements. The most common applied nanoparticles are metal nanoparticles, which are typically synthesized by chemical reduction of the corresponding transition metal salts in the presence of a stabilizer promoting the synthesis of nanoparticles with high stability, rich linking chemistry and solubility [68,69,70,71,72].

#### 2.2.1. Gold Nanoparticles

Gold nanoparticles (AuNPs) are one of the most frequently applied nanomaterials in the biosensing field due to their inherent characteristics such as extraordinary optical and electronic properties, controllable morphology and size, and simple preparation methods [73,74]. In addition, AuNPs exhibit high chemical stability, excellent conducting capability and catalytic activity, large surface area and high surface-to-volume ratio, biological compatibility, and ease of functionalization, favoring the immobilization of biological recognition elements as DNA, antibodies and enzymes [27,32,35,37,73] (Table 2). These features make AuNPs an outstanding nanomaterial for bridging the biological elements with the transduction systems [74].

AuNPs have been extensively used for the development of immuno- and DNA-biosensors for detection of AD biomarkers, being the nanomaterial more frequently reported throughout the reviewed studies. Being very versatile, AuNPs have been applied with different and complementary functions, such as promotion of the conductivity and electrochemical activity of the transducer, immobilization of the biological recognition elements for direct/sandwich detection or conjugated with other nanomaterials. The most common applications of AuNPs were with self-assembled monolayers (SAM), either by being deposited on a SAM-modified platform or serving as the platform for promoting the chemical modification with different SAMs [75,76,77,78], and as amplifying agents modified with secondary antibodies and labels for sandwich immunoassays [46,79,80,81,82]. Finally, AuNPs have also been applied in an isolated manner to transducer surfaces [83,84,85] or in conjugation with other materials such as polyethylene glycol (PEG) [86], MWCNTs [46], and ionic liquid [87]. The published studies clearly acknowledge the vast scope of AuNPs application.

**Figure 7:**
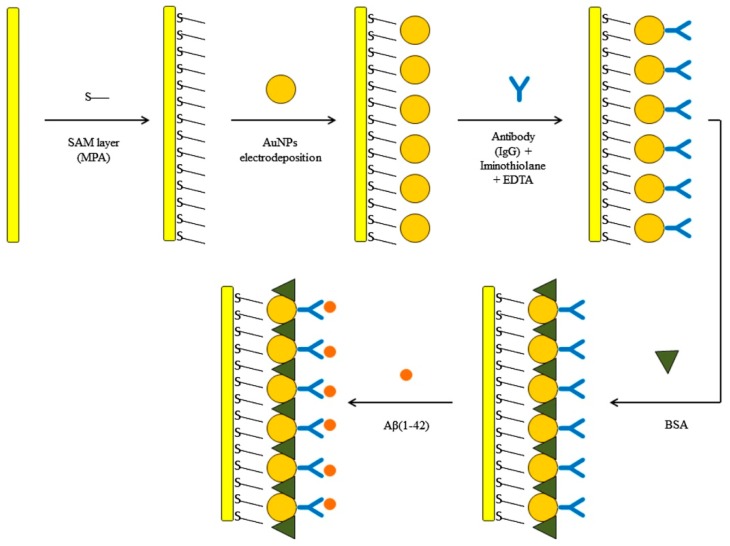
Schematic illustration of the electrochemical immunosensor development using a layer-by-layer approach (Reproduced with permission from [78]. Elsevier, 2017).

Carneiro et al. [78] developed a label-free electrochemical immunosensor based on AuNP-modified gold electrode for Aβ42 determination (Figure 7). In this work, the transducer surface was primarily modified with a mercaptopropionic acid SAM in which AuNPs were electrodeposited. AuNPs were synthesized and deposited simultaneously by applying a negative potential to the previously mercaptopropionic acid modified gold electrode while the electrode was in contact with a chloroauric acid solution. In this study, AuNPs performed a dual role of increasing the electrochemical activity of the transducer while providing an appropriate environment for the immobilization of antibodies. It was possible to observe through square-wave voltammetry (SWV) and electrochemical impedance spectroscopy (EIS) the significant enhancement in the electron transfer process, which had been greatly hindered after the SAM formation, once AuNPs were electrodeposited [78]. In addition, chemically modified antibodies were covalently immobilized on the AuNPs surface through thiol groups introduced into the antibody structure via a thiolation process. With the developed immunosensor, Aβ42 was determined in the range of 10–1000 pg mL^−1^ while attaining a LOD of 5.2 pg mL^−1^ [78]. Further studies concerning selectivity and analytical performance in real samples need to be conducted. 

Hu et al. [84] proposed a colorimetric sandwich immunosensor for Aβ42 detection where AuNPs were individually conjugated with C-terminal antibody and/or N-terminal antibody, to which albumin from bovine serum was added to minimize non-specific adsorptions [84]. Subsequently, Aβ42 peptide was simultaneously captured by the previously formed bioconjugates causing the aggregation of AuNPs, which were accompanied by a color change from red to blue [84]. In this way, taking advantage of the optical properties of AuNPs and via UV-vis spectrometer analysis, Aβ42 concentrations were quantified in the range 7.5–350 nM with a LOD of 2.3 nM while displaying good selectivity and analytical behavior in CSF [84]. On the other hand, further tests concerning the stability parameter need to be conducted.

**Figure 8:**
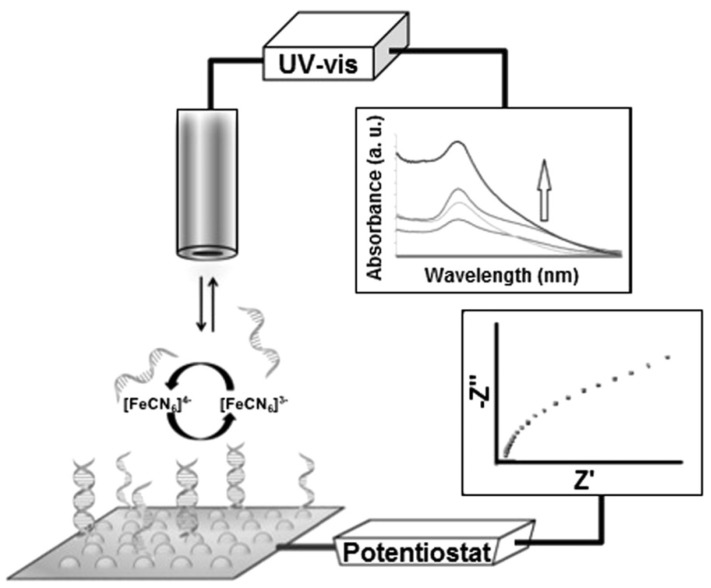
Illustration of the DNA sensor with dual-detection platform for localized surface plasmon resonance (LSPR) and electrochemical impedance spectroscopy (EIS) measurements (Reproduced with permission from [85]. Elsevier, 2014).

Applying a dual detection platform, Cheng et al. [85] developed a biosensor to detect DNA hybridization related to a specific point mutation of ApoE (Figure 8). The platform was based on an ITO modified with electrodeposited AuNPs where DNA probes were immobilized. The electrodeposition process was carried out through cyclic voltammetry, with all recordings being conducted with a constant potential range while the ITO surface was immersed in a chloroauric acid solution leading to the synthesis of AuNPs with 50 to 80 nm. Then, DNA strands were immobilized on the surface of AuNPs causing a shift in peak wavelength in the localized surface plasmon resonance (LSPR) spectra as a result of the effective thickness of the adsorbate layer and electromagnetic decay length. LSPR signal increased in a concentration-dependent manner as target DNA hybridized enabling a 512 nM LOD [85]. Applying EIS to the detection of DNA hybridization and taking advantage of the high conductivity of AuNPs, it was possible to attain a LOD of 286 nM as the immobilization of DNA probe and target led to an increase in the impedance of the platform as a result of the formation of a biological barrier on the AuNPs surface which hindered the electron transfer process [85]. Regardless of displaying good LODs and selectivity for LSPR and EIS, further studies need to be conducted in order to evaluate the reproducibility, stability and analytical behavior in real samples.

Kim et al. [86] developed a shape-code nanoplasmonic biosensor for the determination of three AD biomarkers. The highly selective biosensor was based on a single platform modified with AuNPs of different shapes and antibodies that were analyzed through LSPR. Diverse optical properties can be promoted by the production of AuNPs with different shapes and sizes. As plasmon resonance wavelength is dependent on shape, size and local refractive index surrounding the nanomaterial, the synthesis of AuNPs with different shapes and sizes led to the production of nanoparticles with individual properties that when analyzed through optical spectrometers would function as a bar-code [86]. Three different types of AuNPs were explored, such as spheres (diameter of 50 nm), short rods (aspect ratio of 1.6) and long rods (aspect ratio of 3.6) for detection of Aβ40, Aβ42 and Tau protein, respectively. AuNPs were synthesized via citrate reduction and further modified with PEG with different molecular weights where the antibody immobilization was promoted via an EDC/NHS interaction. With this biosensing configuration, and based on its colorimetric features, multiplexed detection of biomarkers from a single sample was performed, attaining LODs of 34.9 fM, 26 fM and 23.6 fM for detection of Aβ40, Aβ42 and tau protein, respectively [86]. The work developed by Kim et al. [86] was the only one to simultaneously and separately determine three of the most important AD biomarkers with extremely low LODs in addition to successfully measure their levels in samples of human plasma. Nonetheless, studies concerning the biosensor’s stability still need to be conducted.

#### 2.2.2. Other Nanoparticles

AgNPs [88], magnetic nanoparticles [82,89,90] and QDs [48,90] have also been used in the development of biosensors for AD diagnosis but to a lesser extent (Table 2). 

**Figure 9:**
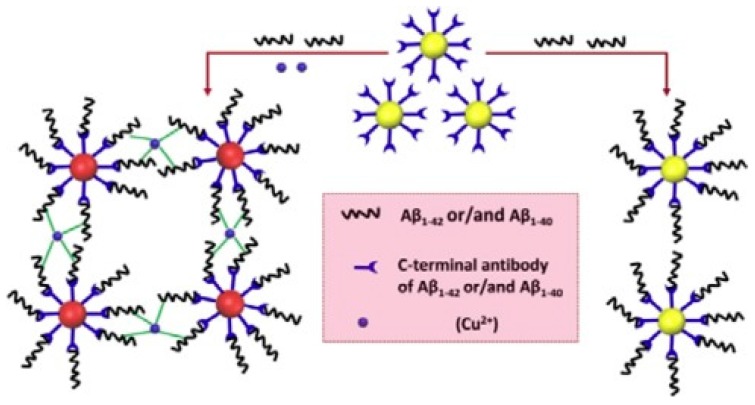
Colorimetric immunosensor for determination of Aβ(1–40/1–42) based on the interaction of Aβ with Cu^2+^ (Reproduced with permission from [88]. Elsevier, 2016).

Presenting characteristics of noble metals such as high conductivity, enhanced electrochemical signal and excellent biocompatibility, AgNPs and their composites have also been explored for the development of biosensing platforms for the determination of several analytes [27,35,91]. In a study developed by Hu et al. [88], a colorimetric immunosensor based on silver nanoparticles was developed for quantification of the Aβ40/Aβ42 ratio (Figure 9), following a similar procedure as the one based on AuNPs for Aβ42 [84]. In this work, the biosensor was based on the interaction between Aβ and Cu^2+^, using AgNPs conjugated with C-terminal antibodies. In the presence of Cu^2+^ and Aβ40/Aβ42 (ratio of 6:1), the conjugate antibody-AgNPs showed high sensitivity towards the analytes of interest leading to the aggregation of modified AgNPs upon binding between Aβ and Cu^2+^, which was accompanied with color change from yellow to red (Figure 9). Through absorbance determination, the immunosensor displayed better sensitivity and selectivity than the previously reported study as a result of AgNPs properties, including distance-dependent color and high extinction coefficient, attaining a LOD of 86 pM [88]. In addition, the colorimetric immunosensor was successfully applied in spiked blood samples.

Magnetic nanoparticles have been the focus of a vast number of studies as a result of their special properties, which include ease of size control, low-cost production, physico-chemical stability, biocompatibility and easy manipulation with magnetic fields, and they are applied in a broad range of biomedical areas as cell labeling, medical imaging, drug delivery, hyperthermia treatment and biosensing [32,35,37,92,93]. Magnetic nanoparticles can be classified as metals, alloys or oxides and are generally based on elements such as iron, cobalt, nickel or manganese, among others [32,93]. These nanoparticles exhibit magnetic behaviors that can be influenced by their small size and shape [32]. When small enough, magnetic nanoparticles can express a superparamagnetic behavior, meaning that magnetization can randomly flip direction under the influence of temperature on reduced time periods, called Neel’s relaxation time [32,37]. This temperature effect disappears in the presence of an external magnetic field becoming magnetized and aligned with the direction of the field but magnetization appears on average zero once the external field is removed [32,37]. Such superparamagnetic behavior prevents agglomeration of the nanoparticles in the absence of a magnetic field, which becomes particularly relevant in specific applications such as magnetic hyperthermia [32]. Moreover, these nanoparticles must be functionalized with hydrophilic and biocompatible coating to prevent aggregation in aqueous solutions while providing a suitable matrix for immobilization of biological recognition elements [32,37].

**Figure 10:**
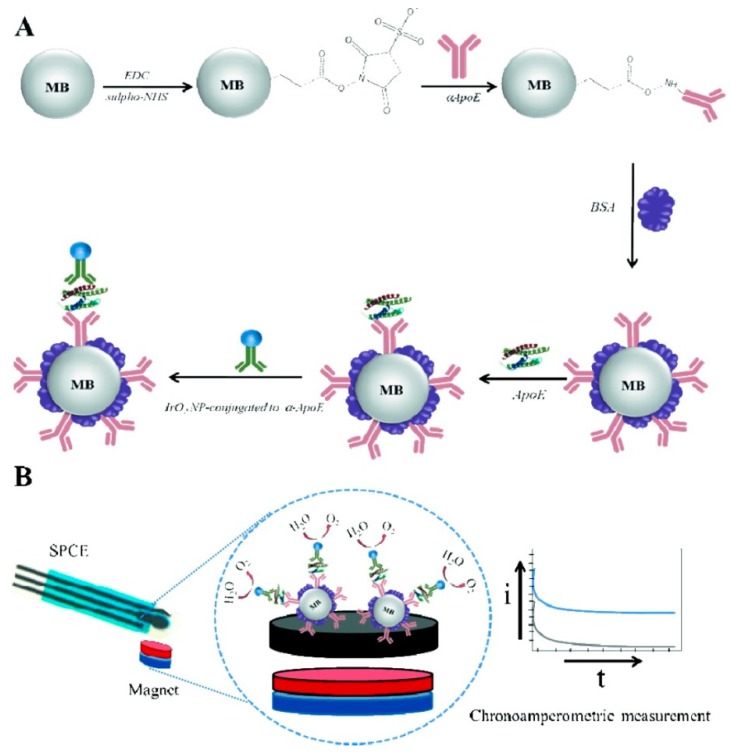
Schematics of the developed experimental procedure: (**A**) magneto sandwich immunoassay using iridium oxide nanoparticles tags and (**B**) electrochemical detection procedure based on the electrocatalytic water oxidation (Reproduced with permission from [89]. John Wiley and Sons, 2014).

De la Escosura-Muñiz [82] developed a biosensor based on a magneto sandwich immunoassay using porous magnetic microspheres (PMMs) as platforms for the recognition event of Aβ and ApoE from human samples. AuNPs were further applied as electrocatalytic agents to complete the sandwich immunoassay and obtain the analytical signal. PMMs were prepared by a multistep swelling polymerization with iron oxide precipitation and combining their high functionality with their large porosity, PMMs offered a high active area that allowed the efficient capture of the target analytes while promoting an enhanced catalytic activity of AuNPs [82]. Antibodies were covalently immobilized on the PMMs surface via EDC chemistry between the carboxylic groups of PMMs and antibody amine groups while the secondary antibodies were randomly adsorbed on the AuNPs surface. The magnetic conjugate was further immobilized on the surface of a screen-printed carbon electrode focusing the electrochemical determination of AD biomarkers on the electrocatalytic behavior of AuNPs towards the hydrogen evolution reaction, which through chronoamperometry attained LODs of 19 pg mL^−1^ and 80 pg mL^−1^ for determination of Aβ and ApoE, respectively [82]. Taking advantage of the excellent structure of PMMs for antibody immobilization, the developed immunosensor was successfully applied in real clinical CSF, serum and plasma samples of patients suffering from AD. Rivas et al. [89] explored the excellent electrocatalytic activity of iridium oxide nanoparticles towards the water oxidation reaction and employed this as a new signaling mechanism in protein diagnosis (Figure 10). The procedure is similar to that previously described by de la Escosura-Muñiz [82] but, instead of AuNPs, the team used iridium oxide nanoparticles. Magnetic beads were modified with antibodies and applied as platforms for immunoassay detection of ApoE [89]. Therefore, antibodies were immobilized on the magnetic beads surface through EDC chemistry while the immobilization of secondary antibodies on iridium oxide nanoparticles was performed by direct random adsorption. The immunocomplex was formed in solution, taking advantage of the great properties of magnetic beads for capturing the analyte and minimizing matrix effects [89]. Then, the secondary antibodies-iridium oxide nanoparticles conjugate was able to recognize ApoE, completing the magneto sandwich immunoassay [89]. Finally, the sandwich immunocomplex was immobilized on the surface of a screen printed carbon electrode where the catalytic activity of the iridium oxide nanoparticles was monitored through chronoamperometry enabling the quantification of ApoE concentration in the range 100–1000 ng mL^−1^ with a 68 ng mL^−1^ LOD in addition to performing ApoE detection in human plasma [89]. The detection based on water oxidation reaction is a simple and sensitive methodology that opens the possibilities for further applications in integrated systems such as those based on lab-on-chip or lateral flow platforms.

Medina-Sánchez et al. [90] developed an on-chip magneto-immunoassay for ApoE via electrochemical detection and using QDs as labels. QDs are luminescent semiconducting materials composed of elements from the groups II–VI or III–V, which have increasingly gained relevance in the development of bioanalytical techniques due to their unique and superior optical and electronic properties, broad absorption spectra, narrow and size-tunable emission spectra, distinctive photoelectrochemical activity and excellent photostability against photobleaching [32,37,94,95]. The possibility of controlling the range of emission wavelengths through changes in QDs sizes opens up the possibility for multiplexed analysis, particularly in the case of optical transduction system, which makes this nanomaterial particularly attractive for application in the diagnosis of most diseases [37,95]. Nonetheless, QD surfaces can be diversely modified either through conjugation with other semiconducting materials with wider band gaps in order to passivate structural defects and enhance quantum yields and photo-stability or with inert and biocompatible coatings to reduce any possibility of toxicity and promote their functionalization with biological recognition elements [32,37,94]. Therefore, Medina-Sánchez et al. [90] developed an immunocomplex based on a microfluidic chip with integrated screen-printed electrodes functionalized with magnetic beads and using QDs as amplifying agents. Firstly, magnetic beads functionalized with antibodies were immobilized on the working electrode through a neodymium magnet placed underneath the substrate. Then, the analyte was flushed through the magnetic beads at different concentrations, followed by detection of biotinylated secondary antibodies. Finally, streptavidin-QDs were introduced to complete the sandwich immunoassay. Electrochemical detection of CdSe@ZnS QDs was conducted through square-wave anodic stripping voltammetry which produced an analytical signal from the reduction of Cd^2+^ to Cd and its further reoxidation [90]. The QDs concentrations were directly related to the levels of ApoE enabling its determination in the range 10–200 ng mL^−1^ with a 12.5 ng mL^−1^ LOD along with performing its detection in human plasma [90]. The use of pH 3.0 buffer in the last step to perform the measurements led to a degradation of the microfluidic platform and SPCE after 15 measurements which makes this platform unreliable for repeated measurements.

### 2.3. Polymer Nanomaterials

Polymers, both natural and synthetic, have been widely used for the development of biosensing platforms due to their easy functionalization and long-term stability [35]. Natural materials, of plant and animal origin, exhibit important characteristics, such as biocompatibility, bioactivity and possibility of biodegradation, allowing them to be successfully applied at a clinical level [96]. On the other hand, the use of synthetic polymers has become an integral component of our everyday life as a result of their chemical resistance, tunable properties and mechanical durability [97]. 

Biosensing technology based on polymer nanomaterials has been commonly employed due to their easy functionalization with biomolecules, high sensitivity and selectivity, hysteresis, and long-term stability [35]. The most common polymeric materials used for the development of biosensing technology for determination of AD biomarkers, through DNA and antigens detection, have been based on homo- and co-polymers [86,98,99,100,101,102], as well as conducting polymers (CPs) [103] (Table 3).

A label-free impedimetric biosensor was proposed by Rushworth et al. [99] for determination of Aβ oligomers based on screen-printed gold electrodes (SPGE) modified with co-polymer of polytyramine/3-(4-hydroxyphenyl) propionic acid. Using a layer-by-layer approach, the selected co-polymer was electropolymerized on the surface of the SPGE followed by conjugation of the biological recognition element via a biotin/neutravidin interaction [99]. The biological recognition element used in this work was the fragment of the cellular prion protein (PrP^c^, residues 95–110), a highly expressed synaptic protein, which mediates the neuronal binding and toxicity of Aβ oligomers [99]. In this work, poly(tyramine) was conjugated with 3-(4-hydroxyphenyl) propionic acid to favor the presence of negatively charged groups that contributed to minimize the non-specific binding of molecules [99]. Through EIS, Aβ oligomers levels were able to be determined in the range 10^−6^–10^−12^ M, attaining a LOD of 0.5 pM [99]. The developed biosensor presented the advantage of being a label-free detection system capable of distinguishing between Aβ monomers and oligomers at an Aβ concentration of 100 pM. Even so, this study still needs to be further validated in real samples.

Conducting polymers have also been applied towards the development of biosensing platforms taking advantage of their high surface area, small dimensions, inherent electrical conductivity and unique physical properties, while also having potential applicability in flexible/wearable electronics in which conducting polymers can display a flexible organic conductor or semiconductor behavior [35,104,105]. In this way, Qin et al. [103] developed a biosensor for monitoring early diagnosis of AD through Aβ oligomers determination based on the electrically conducting poly(pyrrole-2-carboxylic acid) (PPy) and using PrP^c^ as the biological recognition element. The linking agents that promote the immobilization of PrP^c^ on the transducers surface are relatively big molecules that can hinder the electron transfer process, causing significant resistance and, ultimately, compromising the detection of Aβ oligomers at low levels [103]. Therefore, conductive PPy containing carboxylic groups was electrochemically deposited on a gold surface in order to ensure a good electrochemical behavior of the transducer by scanning the electrode from −0.3 to 1.5 V [103]. For immobilization of the biological recognition elements, PrP^c^ was amine functionalized and added to the previously activated PPy modified gold electrode. With the proposed biosensor, Qin et al. [103] were able to attain a 10^−4^ pM LOD while quantifying Aβ oligomers concentrations in the range 10^−7^–10 nM, proving the excellent properties of PPy as an electroactive material and bioreceptor [103]. This study exhibited an exceptionally low LOD (while attaining recoveries of 98–116% in brain samples), a clear selectivity towards Aβ oligomers (when compared with Aβ monomers and fibrils) and high stability after one month at 4 °C.

## 3. Future Perspectives

An early assessment of changes in AD biomarker levels is an essential contribution to informed decisions and the conception of better treatment plans, in addition to increasing the possibilities of success of disease-modifying drugs [10,13,106,107]. Nowadays, three core AD CSF biomarkers have proven diagnostic accuracy for mild cognitive impairment and dementia due to AD, namely Aβ42, total tau, and tau phosphorylated at threonine 181 [10,13,107]. Growing evidence supports the use of the concentration ratio of Aβ42 to Aβ40 as an analytical parameter to improve the accuracy of AD diagnosis when compared to isolated determination of Aβ42, with its main disadvantage being related to an increase in the costs of analysis [13]. In addition, both CSF total and phosphorylated tau are essential for a differential AD diagnosis, underlining the role of phosphorylated tau, since its levels in other dementias are normal [10].

Based on this review, it is possible to outline some serious challenges concerning the development of biosensing platforms in a routine and scalable manner as these devices usually struggle with stability and reproducibility issues when applied to biological samples. In addition, only some studies used real samples obtained from AD patients, with most biosensors being tested in buffer, artificial samples, or real samples spiked with the analytes of interest. On the other hand, the studies that applied the developed biosensors in real samples were able to recognize a clear difference in protein levels between AD and normal individuals [45,46,102,103]. Moreover, particularly in the case of AD, multiplexing detection is a crucial point that is still lacking, since this disease arises from a combination of multiple pathological pathways, ideally requiring the detection of a group of biomarkers in order to ensure a sensitive and accurate diagnosis. Nanomaterials are playing a pivotal role in solving these problems, as nanomaterials with varied sizes display different properties, which translates into differentiated electrochemical and optical behaviors that in turn, when conjugated with specific biological recognition elements, can contribute to differentiated diagnosis. 

## 4. Conclusions

The aging of the world’s population is directly related to the increase in the prevalence of AD, as this disease frequently manifests in the late stages of life. In addition, this neurological disorder can impose a great financial burden on society, as this condition extends through years, highlighting AD as one of the biggest medical and societal challenges of this century. Therefore, there is an urgent need for the development of diagnostic tools that will contribute to more accurate and sensitive diagnosis in the earlier stages of the disorder, also giving a better chance to therapeutics of slowing or stopping disease progression. During the last decade, significant efforts were done towards the development of immuno- and DNA-biosensors for the determination of AD main biomarkers. Sensitivity, selectivity, stability, speed, simplicity and cost benefits continue to be the main parameters for ensuring the quality and performance requirements for the development of novel diagnostic technologies. In this way, the synergy between nanomaterials, biological recognition elements and different transduction techniques has been contributing for the development of biosensors that meet the required parameters. In fact, the outstanding advances in nanotechnology, namely in the synthesis and production of a vast number of nanomaterials, has led to striking developments in the biosensing field. Biosensing platforms coupled with nanostructures not only exhibit a better electrochemical and optical behavior as they also provide an appropriate and biocompatible environment for the immobilization of recognition elements, which is a crucial stage in the development of immuno and DNA biosensors. Thus, throughout this review, the crucial functions that nanomaterials have adopted in the development of biosensing platforms when coupled with different transduction structures were addressed. The most commonly reported nanomaterials for the development of biosensing platforms were AuNPs, carbon nanomaterials (CNTs and graphene), and polymers, and these displayed a variety of functions, but most frequently that of contributing to the enhancement of the biosensor performance due to their excellent conductive and optical properties, which in conjunction with their ease of functionalization promoted stable and oriented immobilization of antibodies and DNA strands on the nanomaterials surface. In addition, nanomaterials were also commonly used as amplifying agents, usually modified with secondary recognition elements and labels, to perform indirect detection of target proteins, which can also contribute to more sensitive and selective analytical procedures.

Apart from the increasing relevance achieved by nanomaterials in the development of immuno- and DNA-biosensors with enhanced performance, there are still some important challenges to overcome before commercialization of biosensing devices with clinical application becomes a reality.

## Figures and Tables

**Table 1:** Biosensors based on carbon nanomaterials for determination of Alzheimer’s disease (AD) biomarkers.

Transducer	Detection Technique	Analyte	Sample	Limit of Detection (nM *)	Ref.
**Carbon Nanotubes**					
CNTs-MESFET/Au strip/Antibodies	Electrical conductance	Aβ42	Human Serum	2.2 × 10^−4^ *	[44]
GCE/SWCNTs-ABTS-PDDA/NKB	DPV	Cu^2+^ Aβ42	Buffer, Blood and Hippocampus of rats	Cu^2+^—40 Aβ42—0.11 *	[45]
GCE/MWCNTs/AuNPs/Gelsolin/Analyte/AuNPs-Gelsolin-HRP	DPV	Aβ40/Aβ42	Buffer, CSF and brain tissue of rats	0.028	[46]
Prism/Au/Antibodies/Analyte/MWCNTs-secondary antibodies	SPR	Tau protein	aCSF	0.125	[47]
**Graphene**					
ITO/graphene-QDs/curcumin/DNA probe	DPV and Fluorescence detection	ApoE4 DNA	Buffer and Human blood plasma	DPV—2.18 ** Fluorescence detection—12.4 **	[48]
GCE/GSHs/DNA probe	DPV	ApoE DNA	Buffer	1 × 10^−5^	[49]
FET/rGO/Antibodies	Impedance	Aβ	Buffer	1 × 10^−6^	[50]

* Value expressed in μg mL^−1^/ng mL^−1^/pg mL^−1^ and converted to nM. ** The results were not possible to convert into molar units, and are thus displayed in pg mL^−1^. aCSF, Artificial cerebrospinal fluid; CNTs-MESFET, Carbon nanotubes film-based metal semiconductor field effect transistor; CSF, Cerebrospinal fluid; DPV, Differential pulse voltammetry; FET, Field effect transistor; GCE, Glassy carbon electrode; GSHs, Mesoporous graphene silica hybrids; HRP, Horeseradish peroxidase; ITO, Indium tin oxide; MWCNTs, Multi-walled carbon nanotubes; NKB, Neurokinin B; QDs, Quantum dots; rGO, Reduced graphene oxide; SPR, Surface plasmon resonance; SWCNTs-ABTS-PDDA, 2,2′-azinobis-(3-ethylbenzothiazoline-6-sulphonate)-poly(diallyldimethylammonium chloride)-bi functionalized single-walled carbon nanotubes composite.

**Table 2:** Biosensors based on nanoparticles (AuNPs, AgNPs and magnetic particles) for the determination of AD biomarkers.

Transducer	Detection Technique	Analyte	Sample	Limit of Detection (nM *)	Ref.
**Gold Nanoparticles**					
SPCE/AuNPs/PEG-MPA/Antibodies/Analyte/Secondary antibodies-ALP	DPV	Aβ42	Buffer, human serum and Plasma	1 × 10^−4^	[75]
Carbon printed chip/AuNPs/MHDA SAM/Protein G/Antibodies	EIS	Aβ42	Buffer	0.57	[76]
AAO/Au film/AuNPs/MUA SAM/Antibody	EIS	Aβ42	Buffer	2.2 × 10^−6^ *	[77]
AuE/MPA SAM/AuNPs/Antibodies	SWV	Aβ42	Buffer	1.15 × 10^−3^ *	[78]
Silicon Wafer/Au/ODT SAM/Antibody fragments/Analyte/AuNPs-antibodies	STM	Aβ42	Buffer	2.2 × 10^−6^ *	[79]
AuE/MPA SAM/Antibodies/Analyte/Aptamer-CS-AuNPs conjugate	DPV	Tau protein-381	Buffer and Human serum	4.2 × 10^−4^	[80]
AuE/MPA SAM/Antibodies/Analyte/Aptamer-CS-AuNPs conjugate	DPV	Tau protein-381	Buffer and Human serum	4.2 × 10^−4^	
AuE/MPA SAM/Antibodies/Analyte/Aβ(1–16)-heme-AuNPs	CV	Aβ40/Aβ42	Buffer and aCSF	0.01	[81]
SPCE/AuNPs/Analyte/Antibodies/ALP-Antibodies	CV	Aβ42	Buffer	0.022 *	[83]
AuNPs/Antibodies/Analyte	Colorimetric UV-Vis	Aβ42	Buffer and Serum samples	2.3	[84]
ITO/AuNPs/Oligonucleotides	LSPR and EIS	ApoE DNA	Buffer	LSPR: 512 EIS: 286	[85]
Glass/APTES/PEG-AuNPs/Antibodies	LSPR	Aβ40 Aβ42 Tau Protein	Dulbecco’s PBS mixed with human plasma samples	Aβ40 3.49 × 10^−5^; Aβ42 2.6 × 10^−5^; Tau protein 2.36 × 10^−5^	[86]
ITO/APTMS/Glutaraldehyde/Ionic Liquid (BMIMBF_4_)/Chitosan/AuNPs/Antibodies/Analyte/Au-TiO_2_/GOx/Antibodies	Colorimetric	ApoE	Buffer and Serum	1.2 × 10^−5^ *	[87]
**Silver Nanoparticles**					
AgNPs/Antibodies	Colorimetric UV-Vis	Aβ40/Aβ42	Buffer and Human blood serum	0.086	[88]
**Magnetic Nanoparticles**					
SPCE/PMMs/Antibodies/Analyte/AuNPs-antibodies	Chronoamperometry	Aβ ApoE	Buffer, CSF, serum and plasma samples of AD patients	Aβ 4.2 × 10^−3^ * ApoE 2.4 × 10^−3^ *	[82]
SPCE/MB/Antibodies/ Analyte/IrO_2_ nanoparticles-secondary antibodies	Chronoamperometry	ApoE	Buffer and Human plasma	2 *	[89]
Graphite ink (microfluidic platform)/MB/Antibodies/Analyte/Antibodies/QDs	SWASV	ApoE	Buffer and human plasma	0.37 *	[90]

* Value expressed in μg mL^−1^, ng mL^−1^ or pg mL^−1^ and converted to nM. AAO, Anodic aluminium oxide; aCSF, Artificial cerebrospinal fluid; AD, Alzheimer’s disease; AgNPs, Silver nanoparticles; ALP, Alkaline phosphatase; APTES, 3-aminopropyltriethoxysilane; APMS, 3-aminopropyltrimethoxysilane; AuE, Gold electrode; AuNPs, Gold Nanoparticles; CSF, Cerebrospinal fluid; CS, Cysteamine; CV, Cyclic voltammetry; DPV, Differential pulse voltammetry; EIS, Electrochemical impedance spectroscopy; GOx, Glucose oxidase; ITO, Indium tin oxide; LSPR, Localized surface plasmon resonance; MB, Magnetic beads; MHDA, 16-mercaptohexadecanoic acid; MPA, Mercaptopropionic acid; MUA, 11-mercaptoundecanoic acid; ODT, 1,8-Octanedithiol; PBS, Phosphate buffer saline; PEG, Polyethylene glycol; PMMs, Porous magnetic microspheres; QDs, Quantum dots; SAM, Self-assembled monolayer; SPCE, Screen-printed carbon electrode; STM, Scanning tunnelling microscopy; SWASV, Square-wave anodic stripping voltammetry; SWV, Square-wave voltammetry.

**Table 3:** Biosensors-based polymers for determination of AD biomarkers.

Transducer	Detection Technique	Analyte	Sample	Limit of Detection (nM *)	Ref.
**Homo- and co-polymers**					
Silicon platform/poly(DMA-co-NAS-co-MAPS)/Antibodies/Analyte/Secondary Antibodies/Cyanine 3	Fluorescent detection	Aβ42	aCSF	0.016 *	[98]
SPGE/POPA co-polymer/PrP^C^ (95–110)	EIS	Aβ oligomers	DMSO/F12 medium and Chinese hamster ovary cell line	5 × 10^−4^	[99]
Interdigitated microelectrode/SiO_2_/APMES/Polyvinyl pyrrolidone-aldehyde solution/Sodium borohydride/Glutaraldehyde/Antibodies	Impedance	Aβ42	Buffer and mouse plasma	2.2 × 10^−5^	[100]
Ion concentration polarization-based preconcentration-Interdigitated microelectrode/SiO_2_/APMES/Polyvinyl pyrrolidone-aldehyde solution/Sodium borohydride/Glutaraldehyde/Antibodies	Impedance	Aβ42	Buffer	8.15 × 10^−6^	[101]
Glass slides/Au film/SAM of carboxyl- and hydroxyl-terminated PEG/Antibodies/Analyte/Antibody	SPR	Aβ40/Aβ42	Buffer and CSF	0.02	[102]
**Conducting Polymers**					
AuE/Poly (pyrrole-2-carboxylic acid)/PrP^C^	EIS	Aβ oligomers	Buffer and Brain samples	10^−7^	[103]

* Value expressed in μg mL^−1^/ng mL^−1^/pg mL^−1^ and converted to nM. aCSF, Artificial cerebrospinal fluid; APMES, 3-(ethoxydimethylsilyl) propylamine; AuE, Gold electrode; CSF, Cereborspinal fluid; DMSO, Dimethyl sulfoxide; EIS, Electrochemical impedance spectroscopy; PEG, Polyethylene glycol; poly(DMA-co-NAS-co-MAPS), Ter-copolymer made with an optimized composition of dimethylacrylamide (DMA), 3-(trimethoxysilyl)propyl methacrylate (MAPS) and N-Acryloyloxy succinimide ester (NAS); POPA, Co-polymer of polytyr-amine/3-(4-hydroxyphenyl) propionic acid; SAM, Self-assembled monolayer; SPGE, Screen-printed gold electrode; SPR, Surface plasmon resonance.
